# Trafficking and persistence of alloantigen-specific chimeric antigen receptor regulatory T cells in *Cynomolgus macaque*

**DOI:** 10.1016/j.xcrm.2022.100614

**Published:** 2022-05-11

**Authors:** Gavin I. Ellis, Kimberly E. Coker, Delaine W. Winn, Mosha Z. Deng, Divanshu Shukla, Vijay Bhoj, Michael C. Milone, Wei Wang, Chengyang Liu, Ali Naji, Raimon Duran-Struuck, James L. Riley

**Affiliations:** 1 Department of Microbiology and Center for Cellular Immunotherapies, University of Pennsylvania, Philadelphia, PA, USA; 2 Department of Pathobiology, University of Pennsylvania, Philadelphia, PA, USA; 3 Department of Pathology and Laboratory Medicine, University of Pennsylvania, Philadelphia, PA, USA; 4 Department of Surgery, University of Pennsylvania, Philadelphia, PA, USA

**Keywords:** organ transplant, bone marrow, adoptive T cell therapy, Foxp3, regulatory T cells, Tregs, chimeric antigen receptor

## Abstract

Adoptive transfer of chimeric antigen receptor regulatory T cells (CAR Tregs) is a promising way to prevent allograft loss without the morbidity associated with current therapies. Non-human primates (NHPs) are a clinically relevant model to develop transplant regimens, but manufacturing and engraftment of NHP CAR Tregs have not been demonstrated yet. Here, we describe a culture system that massively expands CAR Tregs specific for the Bw6 alloantigen. *In vitro*, these Tregs suppress in an antigen-specific manner without pro-inflammatory cytokine secretion or cytotoxicity. *In vivo*, Bw6-specific CAR Tregs preferentially traffic to and persist in bone marrow for at least 1 month. Following transplant of allogeneic Bw6^+^ islets and autologous CAR Tregs into the bone marrow of diabetic recipients, CAR Tregs traffic to the site of islet transplantation and maintain a phenotype of suppressive Tregs. Our results establish a framework for the optimization of CAR Treg therapy in NHP disease models.

## Highlights

• Alloantigen Bw6-specific CAR Tregs can be generated from *Cynomolgus macaque* blood
• CAR Treg product maintains suppressor phenotype and antigen-specific function
• *In vivo*, CAR Tregs preferentially traffic to bone marrow of autologous recipient
• Bw6^+^ islet transplant in bone marrow activates CAR Tregs and prolongs persistence

## Graphical abstract

**Figure:**
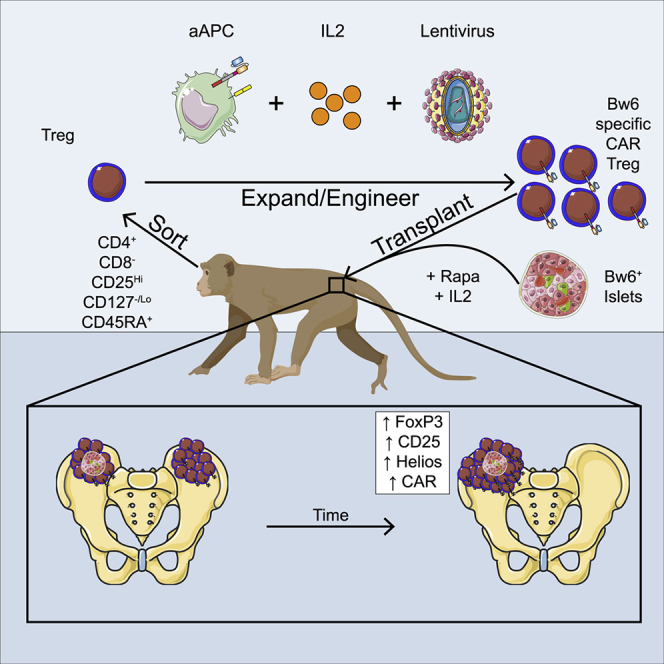

## Teaser

Ellis et al. engineer and massively expand non-human primate alloantigen-specific chimeric antigen receptor (CAR) Tregs. When infused, these CAR Tregs traffic to allogeneic pancreatic islets, become activated, and durably persist in the bone marrow.

## Introduction

The success of solid organ allotransplantation requires sustained curtailment of the immune system’s focus on the destruction of non-self tissue. Despite milestone advancements in tissue procurement, immunosuppression, and surgical technique, 10-year allograft survival rates range from ∼25% to 60%,^1^ and recipients are at increased risk of cancer,^2^ infection,^3^ and organ dysfunction.^4^ These combined factors necessitate the development of novel maintenance immunosuppressive agents that prolong tissue survival and reduce drug-associated morbidity. Regulatory T cells (Tregs) suppress the immune response to maintain immunological homeostasis with self and commensal antigens. As their quantity and function is associated with graft acceptance,^5^^,^^6^ the adoptive transfer of these cells has emerged as a potential therapeutic option.^7^ In phase I clinical trials, polyclonal, non-gene engineered Treg therapy reduced graft-versus-host disease (GVHD) incidence in bone marrow transplant recipients without affecting immune cell reconstitution or graft-versus-leukemia effects.^8^, ^9^, ^10^, ^11^ Treg therapy in solid-organ transplantation is well tolerated and associated with lower rate of infections,^12^, ^13^, ^14^, ^15^, ^16^ allowing for tapering off to tacrolimus monotherapy. However, donor-antigen-specific Tregs show improved immunosuppression of recipient T cells when stimulated by donor cells,^17^, ^18^, ^19^ suggesting that strategies that augment the number of Tregs specific for the allotransplant tissue will be more effective than polyclonal Tregs. Antigen specificity can also be conferred onto Tregs by the engineered expression of chimeric antigen receptors (CARs). CARs typically consist of an antibody-derived single-chain variable fragment (scFv) binder domain fused to T cell co-stimulatory and CD3ζ signaling domains, enabling T cell activation upon binding to a defined cell surface target antigen. For organ transplant, mismatched major histocompatibility complex (MHC) molecules are an attractive target to focus the suppressive properties of CAR Tregs for the following reasons: the transplanted organ would be the only tissue expressing the mismatched MHC, focusing the CAR Tregs to the intended target; MHC class I is highly expressed; and binding MHC triggers no signaling pathways that may alter the function of the transplanted organ.^20^ Indeed, studies in rodents have demonstrated that the adoptive transfer of MHC-class-I-specific CAR Tregs can prevent xenogeneic GVHD^21^ and prolong the survival of xenografts^22^^,^^23^ and allografts.^24^

Non-human primate (NHP) models of transplantation have a long history of translation to humans.^25^^,^^26^ Many of the immunological barriers to long-term allograft acceptance are recapitulated by NHPs, including the presence of pre-exiting alloreactive memory CD8^+^ T cells that drive rejection and are a hallmark of pathogen-educated immune systems.^27^ In addition, the lifespan of NHPs allows for longitudinal studies, and their size allows for transplant to the anatomical location used in human recipients. Because of the close phylogenetic distance between humans and NHPs, many antibody preparations used in transplantation, such as belatacept, anti-thymocyte globulin, and rituximab, cross-react between species, enabling direct assessment in NHPs. CAR binder domains are often cross-reacting, antibody-derived scFvs, alleviating the need to switch to an analogous but disparate scFv for rodent *in vivo* safety and efficacy studies. Since binder domains can influence T cell function through target antigen affinity^28^ and preponderance for tonic signaling,^29^ NHP systems are ideal for the task of direct assessment of safety and efficacy of the exact CAR molecule to be used in human clinical trials.

Thus, NHP models represent a valuable way station on the road to successful clinical translation. Yet, the generation of large doses of engineered NHP Tregs has precluded the evaluation of CAR Tregs in NHP models of transplantation. Therefore, we optimized the manufacturing of human and NHP cross-reactive alloantigen-specific CAR Tregs that retain suppressor function in the absence of cytotoxicity. The infused Tregs remain detectable in peripheral blood transiently and traffic to bone marrow, where they set up residency. Upon adoptive transfer of alloantigen-specific CAR Tregs to a diabetic recipient of a donor islet allograft bearing target MHC antigen, CAR Tregs specifically redistributed to the graft site and displayed an activated phenotype. These studies set the stage for preclinical NHP models of allotransplantation or other immunopathologies.

## Results

### Improved expansion of NHP T cells with aAPCs displaying pan-primate α-CD3 and CD86

**Figure 1:**
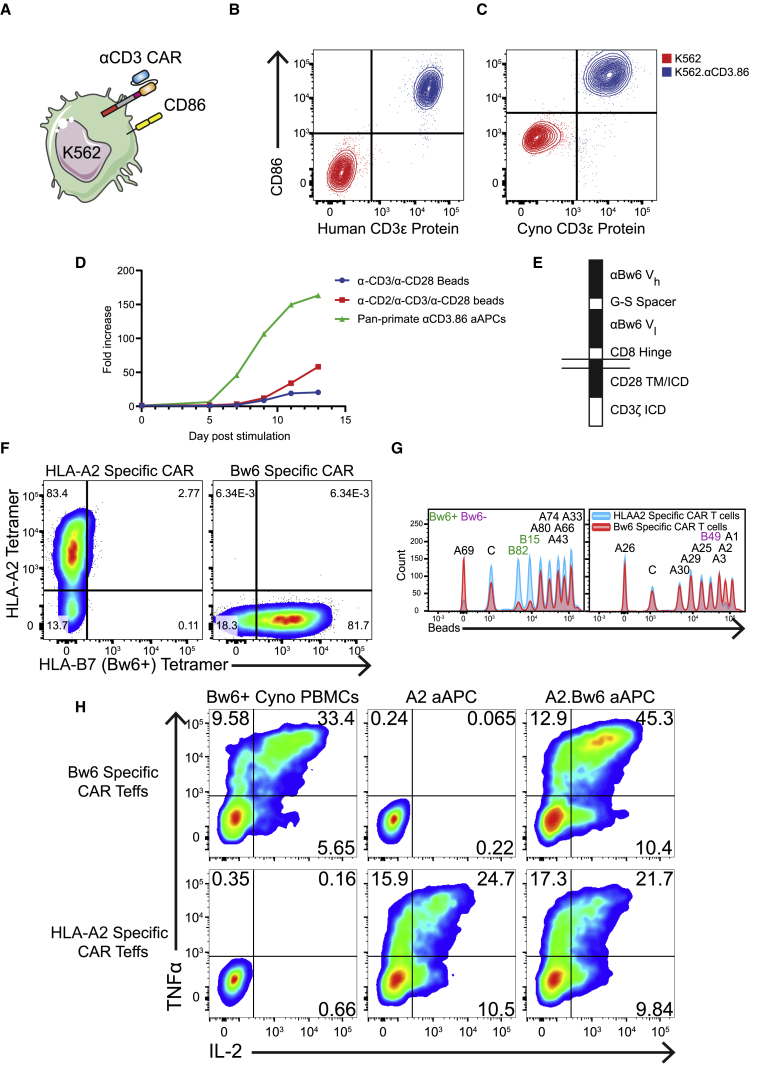
Generation of Bw6-specific CAR Teffs with pan-primate α-CD3 aAPCs (A) Cartoon of aAPCs (K562s) engineered to express αCD3 CAR and CD86. (B and C) Parental and engineered K562 aAPCs were stained with biotinylated, recombinant human CD3ε protein plus streptavidin-PE (B) or with His-tagged *Cynomolgus macaque* CD3ε protein plus α-His antibody (C), followed by α-CD86 antibody. (D) Growth curve of Teffs co-cultured with aAPCs expressing pan-primate αCD3 and human CD86, α-CD3/α-CD28-coated beads, or α-CD2/α-CD3/α-CD28-coated beads. Cells were counted every 2 to 3 days and diluted with media. Data are representative of two independent experiments. (E) Schematic of Bw6-specific CAR. ICD, intracellular domain; TM, transmembrane domain; Vh, antibody variable heavy domain; Vl, antibody variable light domain. (F) *Cynomolgus macaque* T cells were activated with aAPCs and transduced with χHIV lentiviral vectors encoding Bw6-specific CAR or HLAA2-specific CAR and then stained with both HLA-A2 and HLA-B7 (Bw6) tetramers. (G) HLAA2-specific (blue) or Bw6-specific (red) human CAR T cells were incubated with single-antigen FlowPRA beads before analysis on a flow cytometer. Each peak represents beads conjugated to a unique HLA molecule (black, HLA-A molecules; green, Bw6^+^ HLA-B molecules; purple, Bw6^−^ HLA-B molecules). Histograms are gated to depict unbound beads, such that a drop in frequency represents binding to CAR T cells. C, control beads. (H) Human CAR Teffs were co-cultured for 5 h with the indicated target before staining with α-TNFα and α-IL-2 antibodies. Data are representative of three independent experiments.

Expansion kinetics of NHP T cells stimulated with α-CD3/α-CD28-coated magnetic beads exhibit a considerable lag in growth relative to human T cells, forcing extended *ex vivo* culture to achieve therapeutic doses.^30^, ^31^, ^32^ Adherent rodent-based artificial antigen-presenting cells (aAPCs) have shown improved expansion kinetics,^17^^,^^33^, ^34^, ^35^, ^36^, ^37^ but these aAPCs face significant obstacles to be used to expand human T cells for human use. Previously, non-adherent, human cell line (K562) aAPCs^38^, ^39^, ^40^ expressing CD86 and CD64 (to facilitate binding by α-CD3 antibodies) were used to manufacture human T cells with higher cell yields and better function than bead-based approaches.^30^^,^^41^, ^42^, ^43^, ^44^, ^45^ Importantly, a Good Manufacturing Practice (GMP) version of these aAPCs was used to expand human Tregs for human use in a phase I clinical trial where the yield of Tregs was far superior to a similar manufacturing process that used α-CD3/α-CD28-coated beads.^10^^,^^46^ We considered a similar strategy to expand NHP T cells; however, we were unable to find an α-primate CD3 antibody that bound CD64 with high affinity. To overcome this limitation, we identified an scFv that binds primate CD3.^47^ We converted this scFv into a CAR and transduced this molecule into K562 cells previously engineered to express CD86, generating ready-to-use, primate-specific aAPCs to expand T cells (Figures 1A–1C).^42^ In comparison to a bead-based approach, cellular aAPCs induced NHP effector T cells (Teffs) to expand faster and to a greater degree (Figure 1D), making adoptive T cell transfer studies in NHP more feasible.

### Bw6-specific CAR T cells recognize human and NHP Bw6^+^ cells

To model CAR Treg therapy in NHPs, we wanted to identify an alloantigen that could be targeted by a CAR in both NHPs and humans. Bw6 is a “public” MHC epitope identified as a frequent transplant alloantigen due to its inclusion in some, but not all, human leukocyte antigen B (HLA-B) and HLA-C molecules.^48^ Because many NHPs express MHC molecules containing the Bw6 epitope and many do not, it is feasible to identify both donors and recipients for MHC-mismatched tissue transplants. We generated a Bw6-specific CAR by adapting an α-Bw6 antibody into an scFv and then appending human CD8 hinge, CD28 transmembrane, and CD28/CD3ζ signaling domains to the receptor (Figure 1E). As a control, we generated a similar CAR construct that recognizes HLA-A2^49^ and does not cross-react with any known NHP MHC. Binding of HLA-B7 (Bw6^+^), but not HLA-A2, tetramer to Bw6 CAR-transduced human Teffs confirmed expression and trafficking of Bw6 CAR to the cell surface (Figure 1F). To ascertain CAR binding specificity across many MHC molecules, we co-cultured CAR Teffs with HLA single-antigen conjugated beads.^41^ Depletion of beads from the pool of unbound beads revealed CAR binding to HLA-B82 and B15 (Bw6^+^) without binding B49 (Bw6^−^) or any of the HLA-A molecules tested (Figure 1G). Bw6-specific CAR Teffs were able to secrete cytokines in response to Bw6 expressed in NHP and human MHC, but not HLA-A2 (Figure 1H), demonstrating the antigen specificity required for CAR Treg therapy in mismatched allotransplantation.

### Massive *ex vivo* expansion of engineered, allospecific NHP Tregs

**Figure 2:**
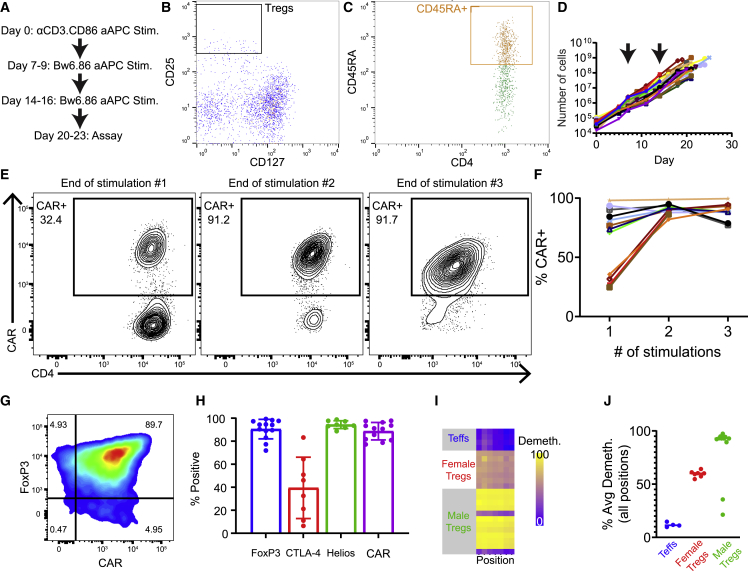
Generation and expansion of *Cynomolgus macaque* CAR Tregs *in vitro* (A–C) Outline of Treg expansion protocol. Freshly isolated PBMCs from *Cynomolgus macaque* were stained with CD4, CD25, CD127, and CD45RA antibodies and flow sorted to obtain the top 1% to 2% of CD25^hi^ CD127^−/lo^ population (B) that are CD45RA^+^ (C). (D) Following sort, irradiated α-CD3.CD86 aAPCs were co-cultured with Tregs at one aAPC per one Treg. After 48 h, Bw6-specific CAR lentiviral vector was added, and the scheme outlined in Figure 3A was followed to expand the Bw6-specific CAR Tregs. Cells were counted every 2 to 3 days, and cell growth was graphed, with each line representing 1 of 19 independent sorts. Arrows indicate days of restimulation with irradiated Bw6.86 aAPCs. (E) Tregs were stained with HLA-B7 (Bw6^+^) tetramer upon resting before each restimulation. Displayed is a representative example showing CAR Treg enrichment after antigen-specific restimulation. (F) Summary data from 12 experiments showing CAR Treg enrichment via restimulation. Each line represents one independent sort and expansion. (G) At the end of expansion, cells were stained for FoxP3 and Bw6-specific CAR expression. (H) Summary of 13 experiments showing the percentage of expanded Bw6-specific Tregs expressing FoxP3, CTLA-4, Helios, and Bw6-specific CAR at the conclusion of expansion. Data are represented as mean ± SEM. (I) Genomic DNA collected at the end of cell culture was assessed for methylation of FOXP3 at the Treg-specific demethylated region (TSDR) by bisulfite sequencing. Each row represents one independent product, and each column represents one CpG locus in the TSDR. (J) Summary of average TSDR demethylation across all loci from 18 experiments with six different animals. Each data point is one Treg expansion, and line represents the group mean.

Having optimized the generation of NHP CAR Teffs, we adapted our protocol to the manufacture of Bw6-specific NHP CAR Tregs (Figure 2A). CD4^+^ CD25^+^ CD127^−/lo^ CD45RA^+^ Tregs were sorted from peripheral blood mononuclear cells (PBMCs) (Figures 2B and 2C) before co-culture with the pan-primate aAPCs developed in Figure 1. Based on previous work characterizing *Cynomolgus macaque* Tregs,^17^ we collected the top 1% to 2% of CD25^+^ Tregs among CD4^+^ T cells. We then further gated on CD45RA^+^ to isolate naive Tregs that maintain FoxP3 expression and suppressor function across multiple rounds of stimulation^50^^,^^51^ and are more prevalent in juvenile versus adult animals (Figure S1). From a single ∼20-mL blood draw, we recovered between ∼10,000 and 100,000 Tregs (mean: 44,354 ± 23,435) that were expanded according to the schedule depicted in Figure 2A. After 2 days in culture, Tregs were transduced with HIV lentiviral vector incorporating a simian immunodeficiency virus capsid,^52^^,^^53^ which outperformed HIV capsid lentiviral vectors in transducing NHP T cells (Figure S2). To select for functional CAR^+^ Tregs, we performed antigen-specific restimulation with aAPCs engineered only with HLA-B7 (Bw6^+^) and CD86, at 7-day intervals. Over a 21- to 24-day culture period, cell populations expanded an average of 11,173- ± 3,122-fold to doses of up to 1 × 10^9^ cells (Figure 2D) with a pronounced enrichment of CAR^+^ Tregs (Figures 2E and 2F). At the conclusion of manufacture, Tregs expressed high levels of FoxP3, Helios, and CAR and variable levels of CTLA-4 (Figures 2G and 2H) but no longer expressed CD45RA (Figure S3). The Tregs also had high demethylation of the Treg-specific demethylated region (TSDR) (Figures 2I and 2J), a measure of stable FoxP3 expression.^54^ Of note, two of our early expansion products demonstrated low FoxP3 expression and suppressor activity, and these two products had highly methylated TSDR, confirming the correlation between demethylation of TSDR and suppressor activity. Subsequent experiments in the same animals using more stringent gating for Tregs resulted in highly suppressive CAR Tregs with demethylated TSDR. Rested Tregs were cryopreserved to allow for pooling of doses and flexibility in the clinical protocol. Thawed Tregs restimulated with Bw6^+^ aAPCs and grown for an additional 7 days retain FoxP3, Helios, and CAR expression and expand a further ∼10-fold (Figure S4).

### Engineered Bw6-specific CAR Tregs demonstrate antigen-specific suppressor function without being pro-inflammatory *in vitro*

**Figure 3:**
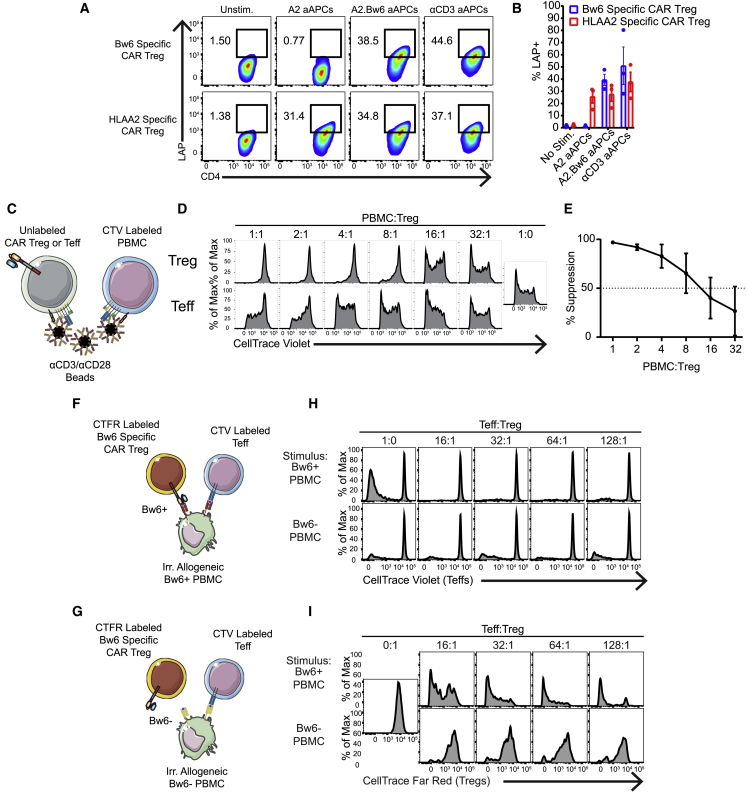
*Cynomolgus macaque* CAR T cells demonstrate antigen-specific suppressor function (A and B) Bw6- or HLA-A2-specific CAR Tregs were co-cultured with the indicated aAPC for 24 h and then stained for LAP expression (n = 3 independent experiments). (C) Antigen non-specific suppression was measured by co-culture of unlabeled Bw6-specific CAR Tregs or Bw6-specific CAR Teffs with CellTrace Violet (CTV)-labeled allogeneic PBMCs and α-CD3/α-CD28 beads at the indicated PBMC:Treg ratio for 4 to 5 days. (D) Histograms depict proliferation of the CD4^−^ CD8^+^ CTV^+^ cells present in the allogenic PBMCs of experiment performed as depicted in (C). (E) Line graph representing mean ± SEM of seven independent experiments performed as in (D). (F and G) Antigen specific suppression was assessed by mixed lymphocyte reaction (MLR). CTV-labeled responder Teffs and CellTrace Far Red (CTFR)-labeled, Bw6-specific CAR T cells were co-cultured with unlabeled, irradiated allogeneic PBMCs from either Bw6^+^ or Bw6^−^ animals. (H) Histograms showing proliferation of the CD4^−^ CD8^+^ CTV^+^ Teffs in the MLR performed as depicted in (F) and (G). (I) Histograms showing proliferation of CD4^+^ CTV^−^ CTFR^+^ Bw6-specific Tregs in same MLR wells as (H). MLRs were assessed between seven pairs of animals in two independent experiments, and similar data were obtained in both experiments.

We next sought to characterize *in vitro* antigen specificity and function of the expanded Bw6-specific CAR Tregs. To demonstrate antigen specificity of expanded CAR Treg product, HLA-A2-specific or Bw6-specific CAR Tregs were mixed with aAPCs expressing HLA-A2 with or without HLA-B7 (Bw6^+^). Following overnight co-culture, Bw6-specific CAR Tregs upregulated latency-associated peptide (LAP), a component of latent transforming growth factor β (TGF-β), only in the presence of target Bw6 antigen (Figures 3A and 3B). The Bw6-specific CAR Treg product could also suppress proliferation of Bw6^−^ bystander PBMCs when Tregs and PBMCs were both non-specifically stimulated through endogenous T cell receptor (TCR) with α-CD3/α-CD28 beads (Figures 3C–3E). To further assess antigen specificity and suppressor function, we performed mixed lymphocyte reactions where CellTrace-Violet-labeled Teffs and autologous, CellTrace-Far-Red-labeled, Bw6-specific CAR Tregs were co-cultured with allogeneic PBMCs from Bw6^+^ or Bw6^−^ NHPs (Figures 3F and 3G). As expected, there was more alloreactive T cell proliferation between Teffs and Bw6^+^ PBMCs than between two Bw6^−^ animals in the unsuppressed condition. However, Bw6-specific CAR Tregs suppressed Teff cell proliferation better when stimulated by Bw6^+^ PBMCs versus Bw6^−^ PBMCs, with greater than 50% suppression at a ratio of one Treg for every 128 Teffs (Figures 3F–3H). In addition, Bw6-specific CAR Tregs only proliferated themselves in response to Bw6^+^ PBMCs (Figures 3F, 3G, and 3I), further demonstrating their antigen specificity.

**Figure 4:**
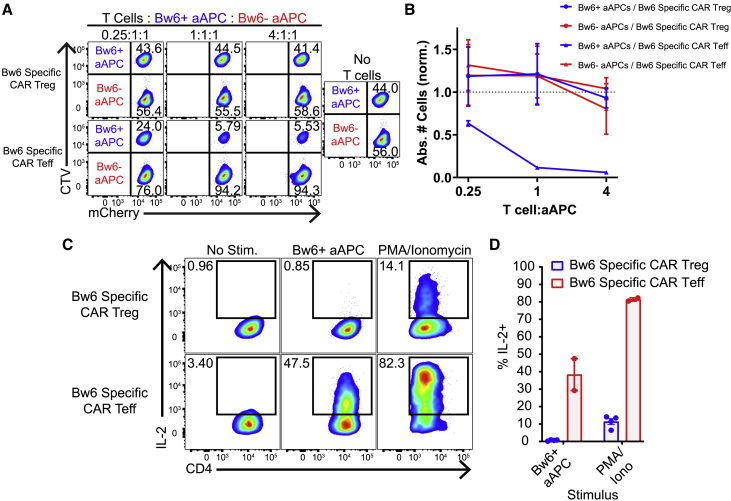
*Cynomolgus macaque* Bw6-specific CAR T cells are not pro-inflammatory (A) Persistence of on-target Bw6^+^ aAPCs and off-target Bw6^−^ aAPCs after 24 h co-culture with Bw6-specific CAR Tregs or Bw6-specific CAR Teffs. aAPCs were transduced with mCherry to distinguish from CAR Teffs or CAR Tregs. (B) Absolute number of aAPCs remaining in (A) were normalized to the number of cells in well with aAPCs alone (n = 2 independent experiments). Data are represented as mean ± SEM. (C and D) IL-2 secretion following 5 h co-culture between indicated cell types (n = 4) is shown. Data are represented as mean ± SEM, with each data point representing one independent experiment.

Some studies have noted the cytotoxic potential of Tregs, which could accelerate graft rejection.^55^ To test whether CAR Tregs kill target cells, we mixed Bw6-specific CAR Tregs or Bw6-specific CAR Teffs with on-target Bw6^+^ aAPCs and off-target Bw6^−^ aAPCs in overnight co-culture. Bw6-specific CAR Tregs did not perturb either the absolute or relative numbers of surviving aAPCs, while Bw6-specific CAR Teffs specifically and significantly reduced on-target cell number (Figures 4A and 4B). This lack of CAR Treg cytotoxicity was confirmed by measuring active caspase 3 expression in Bw6^+^ K562 cells when cultured in the presence of Bw6-specific CAR Tregs (Figure S5). Bw6-specific CAR Tregs did not secrete interleukin-2 (IL-2) in response to target antigen, supporting their non-inflammatory character (Figures 4C and 4D). However, they did make measurable but reduced levels of tumor necrosis factor alpha (TNFα) and MIP1β relative to Bw6-specific CAR Teffs (Figure S6). Overall, the Bw6-specific CAR Treg product is suppressive in response to specific antigen without any cytotoxic or pro-inflammatory character from converted or contaminating T cells and is thus suitable for therapeutic adoptive transfer.

### Adoptive transfer of BW6-specific CAR Tregs into Bw6^+^ NHP resulted in no adverse events

**Figure 5:**
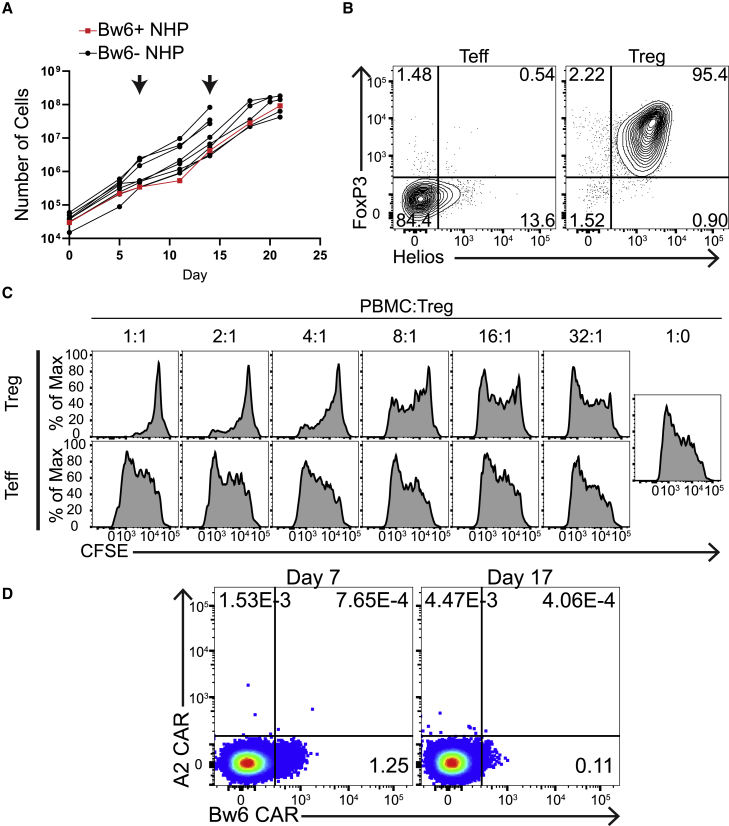
Adoptive transfer of BW6-specific CAR Tregs into Bw6^+^ NHP resulted in no adverse events (A) Growth of Bw6-specific CAR Tregs from Bw6^+^ NHP *in vitro*. Each black line represents one independent expansion of Bw6-specific CAR Tregs from Bw6^−^ NHP, while red line represents growth of Bw6-specific CAR Tregs from Bw6^+^ NHP. Arrows indicate days of restimulation with irradiated Bw6.86 aAPCs. (B) Expression of FoxP3 and Helios at the conclusion of manufacture of Bw6-specific CAR Tregs and CAR Teffs grown from Bw6^+^ animal. (C) Bw6-specific CAR Tregs were co-cultured for 5 days with Carboxyfluorescein succinimidyl ester (CFSE)-labeled allogeneic PBMCs and α-CD3/α-CD28 beads at the indicated PBMC:Treg ratio to assess non-specific suppressor function as in Figure 3C. Histograms depict proliferation of the CD4^−^ CD8^+^ CFSE^+^ cells present in the allogenic PBMCs from Bw6^−^ animal. (D) Following adoptive transfer of Bw6-specific CAR Tregs and HLA-A2-specific CAR Teffs to autologous Bw6^+^ recipient, whole blood was stained with HLA-A2 or HLA-B7 (Bw6^+^) tetramer at indicated time points. Dot plots are gated on CD4^+^ CD8^−^ FoxP3^+^ cells.

On-target, off-tissue toxicity is a major concern for CAR Teff cell therapy.^56^ We wanted to perform a pilot experiment to determine whether on-target, off-tissue toxicity was going to be a significant concern for CAR Treg therapy. We hypothesized that infusing autologous Bw6^+^ CAR Tregs back into its donor would represent the most extreme example of on-target, off-tissue toxicity, since nearly every cell in the body would be recognized by the CAR Treg. To do this experiment, we first had to manufacture Bw6-specific CAR Tregs from a Bw6^+^ animal. Previous studies that tried to expand CAR Teffs specific for markers expressed on T cells resulted in massive fratricide that rapidly eliminated the entire culture.^57^ We did not observe such fratricide-expanding CAR Tregs, in which the target antigen is expressed on all cells in the culture. By restimulating Bw6-specific CAR Tregs from a Bw6^+^ animal with pan-primate α-CD3/CD86 aAPCs on days 7 and 14, we were able to manufacture a product with similar expansion kinetics as Bw6-specific CAR Tregs from Bw6^−^ NHPs, further demonstrating their lack of cytotoxicity (Figure 5A). Bw6-specific CAR Tregs from this Bw6^+^ NHP retained FoxP3 and Helios expression (Figure 5B) and *in vitro* suppressor function (Figure 5C). Autologous Bw6-specific CAR Tregs were co-transferred with HLA-A2-specific CAR Teffs into the Bw6^+^ recipient to assess their relative persistence. We were able to detect Bw6-specific CAR Tregs in circulation for >7 days without detecting any CAR Teffs (Figure 5D), indicating that the antigen-specific Tregs were able to persist in the peripheral blood longer than Teffs expressing a CAR that did not have a target within the NHP. Staining of CAR on Tregs generated from a Bw6^+^ animal was less efficient than those generated from a Bw6^−^ animal, potentially because of epitope blocking in *cis* or internalization of chronically activated CAR. Despite target antigen expression on every MHC^+^ cell *in vivo*, infusion of 6.54 × 10^6^ cells/kg (∼50 million) Bw6-specific CAR Tregs did not result in any overt toxicity at this relatively low cell dose, a positive sign for the safety of using adoptively transferred CAR Tregs to facilitate organ transplant.

### Bw6-specific CAR Tregs preferentially home to bone marrow following adoptive transfer in antigen-negative recipient

**Figure 6:**
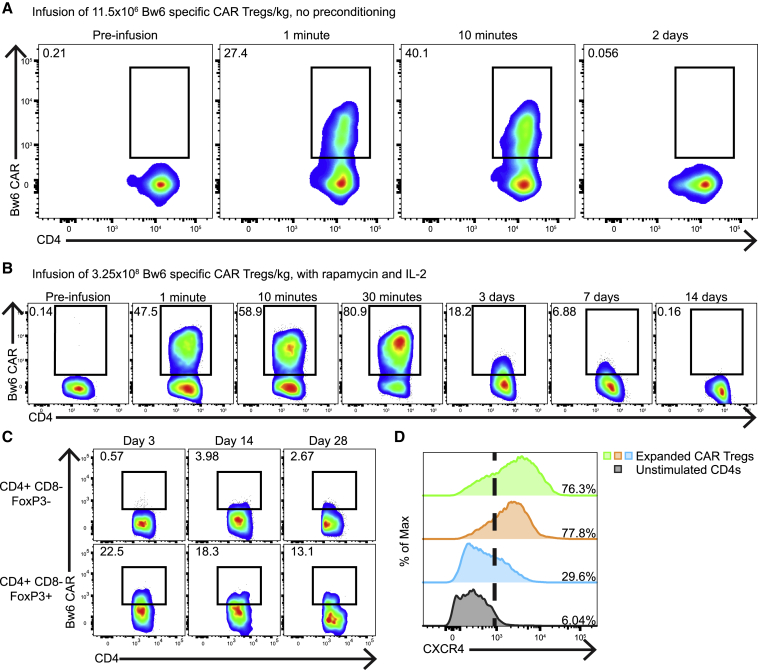
CAR Tregs persist and home to bone marrow following adoptive transfer into antigen-negative host (A) Following adoptive transfer of Bw6-specific CAR Tregs into a non-preconditioned, antigen-negative recipient, peripheral blood was stained with Bw6 tetramer to detect Bw6-specific CAR Tregs. Dot plots show CD4^+^ CD8^−^ FoxP3^+^ gated cells in peripheral blood at the indicated time points. (B and C) Detection of Bw6-specific CAR molecule on CD4^+^ CD8^−^ FoxP3^+^ gated cells in peripheral blood (B) or bone marrow (C) at the indicated time points following adoptive transfer of Bw6-specific CAR Tregs into rapamycin and IL-2 preconditioned, antigen-negative animal. (D) Flow cytometry of CXCR4 expression from three separate Bw6-specific CAR Treg expansion products and unstimulated CD4s.

Having demonstrated *in vitro* function and safety of Treg infusion, we adoptively transferred autologous Bw6-specific CAR Tregs into a Bw6^−^ animal. A mixture of thawed and fresh autologous Bw6-specific CAR Tregs was infused into a Bw6^−^ primate at a dose of 11.5 × 10^6^ cells/kg without preconditioning. Immediately following infusion, Bw6-specific CAR^+^ Tregs were found in peripheral circulation; however, 2 days later, these cells were below the limit of detection (Figure 6A). In a separate study using the same animal after a wash out period of 14 months, we delivered a large dose of 3.25 × 10^8^ Bw6-specific CAR Tregs/kg along with infusions of rapamycin and IL-2, which have been shown to enhance the persistence and stability of adoptively transferred Tregs.^58^ Greater than 80% of all CD4^+^ FoxP3^+^ cells in the peripheral blood were CAR^+^ 30 min post-infusion, but both abundance and mean fluorescence intensity (MFI) of CAR Tregs waned through 7 days post-transfer until falling below the limit of detection by 14 days (Figure 6B). At 28 days post-CAR Treg infusion, the animal was euthanized to investigate the dissemination of engineered Tregs. Though undetectable in lymph nodes, peripheral blood, and spleen, CAR^+^ Tregs were found in bone marrow at all time points (Figure 6C). A retrospective analysis of expanded Tregs determined that many cells expressed the bone marrow homing chemokine receptor CXCR4 (Figure 6D), suggesting that the preferential bone marrow accumulation is likely in both antigen-positive and negative CAR Treg recipients.

### Bw6-specific CAR Tregs traffic to Bw6^+^ allograft in the bone marrow

**Figure 7:**
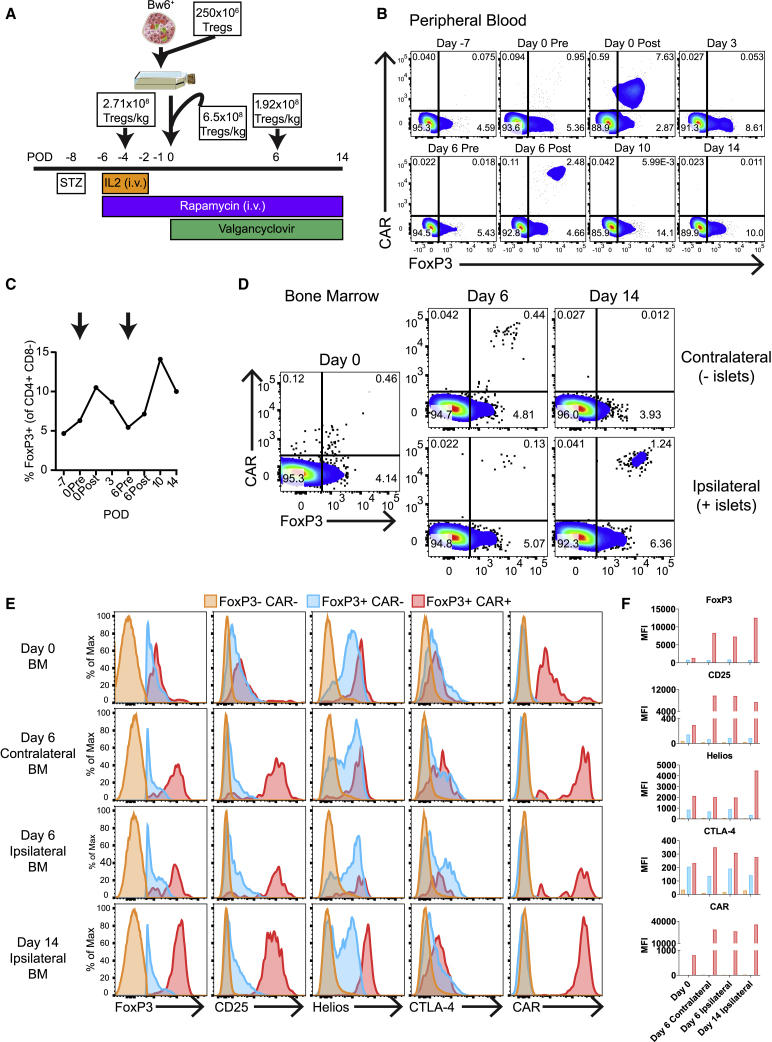
Bw6-specific CAR Tregs traffic to Bw6^+^ allograft in the bone marrow (A) Outline of transplant. On post-operative day (POD) −8, a Bw6^−^ recipient NHP was rendered diabetic by a single dose of streptozotocin (STZ). Four days later, a first dose of Tregs was administered split between the bone marrow and i.v. Donor islets were harvested on POD −1 and co-cultured with Tregs overnight in media with IL-2. The following day, 1.3 × 10^9^ Tregs were added to the islet/Treg mixture and infused into the bone marrow along with 1.3 × 10^9^ Tregs infused i.v. On POD 4 and POD 14, tertiary and quaternary Treg doses were infused i.v. The islet recipient was given IL-2, rapamycin, and valganciclovir as indicated. Plots are gated on CD4^+^ CD8^−^ cells. (B) Peripheral blood was stained with Bw6 tetramer to detect Bw6-specific CAR Tregs at the indicated time points. (C) Percent of CD4^+^ CD8^−^ peripheral blood T cells that expressed FoxP3. Arrows represent infusions of Tregs. (D) Ipsilateral (graft site) or contralateral (non-islet-containing) bone marrow aspirates were stained with Bw6 tetramer at the indicated time points. Plots are gated on CD4^+^ CD8^−^ cells. (E and F) Flow cytometry phenotype of FoxP3^−^ CAR^−^ (orange), FoxP3^+^ CAR^−^ (blue), and FoxP3^+^ CAR^+^ cells (red). (F) MFI of data represented in (E) is shown.

The bone marrow is a well-vascularized space that has been explored as an alternative site for pancreatic islet transplantation in both humans^59^ and NHPs.^60^ Since Bw6-specific CAR Tregs trafficked to the bone marrow in the absence of antigen, we decided to perform intraosseous Bw6^+^ islet allograft transplantation into a diabetic NHP in concert with adoptive transfer of Bw6-specific CAR Tregs as outlined in Figure 7A. Briefly, Bw6-specific CAR Tregs were generated from the recipient NHP’s blood and cryopreserved. The recipient animal was then rendered diabetic with a single injection of streptozotocin (STZ). Because it took about 3 days for Bw6-specific CAR Tregs to traffic to the bone marrow in an antigen-negative recipient (Figure 6C), we first infused our recipient with 2.71 × 10^8^ Tregs/kg supported by daily IL-2 injections^61^ 4 days before transplant as a dose split between intravenous (i.v.) and bone marrow. Bw6^+^ donor islets were then harvested and co-cultured overnight with 250 × 10^6^ Bw6-specific CAR Tregs to allow for pre-activation of the Tregs *in vitro*. The following day, 3.25 × 10^8^ Bw6-specific CAR Tregs/kg were added to the Treg/islet mixture and infused into the iliac crest along with another 3.25 × 10^8^ cells/kg i.v. Six days following transplant, we infused another 1.92 × 10^8^ Bw6-specific CAR Tregs/kg i.v. Flow staining of peripheral blood showed a transient increase in FoxP3^+^ cells following the first two Bw6-specific CAR infusions (Figures 7B and 7C), similar to that seen in antigen-negative animals. Six days after transplantation, there were Bw6-specific CAR Tregs in both the ipsilateral and contralateral bone marrow, but by 14 days post-transplantation, the Bw6-specific CAR Tregs had redistributed entirely to the ipsilateral side (Figure 7D). The CAR^+^ FoxP3^+^ Tregs displayed an activated phenotype, with higher expression of FoxP3, CD25, Helios, and CTLA-4 versus endogenous FoxP3^+^ CAR^−^ Tregs (Figures 7E and 7F). The Bw6-specific CAR T cells also had much higher CAR expression on a per-cell basis (Figures 7E and 7F) versus that of antigen-negative animals in Figure 6C. Altogether, we describe the *ex vivo* manufacturing of engineered CAR Tregs for preclinical testing in NHPs. These CAR Tregs naturally home to the bone marrow and persist for greater than 1 month. Upon introduction of target^+^ allograft tissue, the CAR Treg can further home and concentrate at the site of transplant. These studies now set the stage to determine whether these alloantigen-specific CAR Tregs can induce long-term tolerance to allotransplant.

## Discussion

In this fledgling age of cellular therapies, rodent rather than NHP models have provided the bulk of preclinical efficacy and safety data needed to support phase I human clinical trials, largely due to difficulties manufacturing engineered NHP T cells and modeling tumors in NHPs. However, excellent NHP models exist for therapies aiming to mediate organ transplant tolerance and have been instrumental in developing many current approaches to facilitate organ transplant, including co-stimulatory blockade, and mixed chimerism, as well as immunosuppressive agent dosage and timing.^25^ Here, we show that, from a single venous blood draw, Tregs can be engineered and expanded to generate a near-pure culture of Bw6-specific CAR Tregs suitable for use in preclinical allotransplantation studies. These expanded CAR Tregs are suppressive *in vitro*, retain antigen specificity, and after adoptive transfer to islet transplant recipients, are able to traffic to allograft tissue and persist in an activated state.

While the exact number of Tregs needed to prevent or mitigate alloimmune responses or establish long-term tolerance remains to be defined,^33^^,^^34^ there was a strong correlation between the number of Tregs infused and the absence of GVHD after bone marrow transplant in lymphoma patients receiving double umbilical cord graft.^10^ Moreover, there have been no dose-limiting toxicities observed with polyclonal Treg infusion, suggesting that large numbers of Tregs can be safely delivered. To obtain large number of CAR Tregs is especially challenging relative to generating large numbers of effector T cell populations. For one, Tregs are a rare population that lack unique cell surface markers necessary to obtain a pure population.^62^ In addition, Tregs are hypoproliferative relative to Teff during *ex vivo* culture. Thus, a small contamination of Teff in an initial sorted population can expand significantly during a 2 to 3 weeks culture.^17^ Our approach focused on obtaining as pure population as possible up front using stringent gating to minimize the number of Teffs and maximize the number of naive CD45RA^+^ Tregs, which better maintain Treg identity and function in long-term culture of human cells.^50^ The downside to this approach was a miniscule initial starting population, necessitating a robust Treg expansion system to obtain therapeutic levels of Tregs. aAPCs expressing an α-CD3 CAR and CD86 provide a potent initial stimulation that activates cells sufficiently to increase yield and allow for lentiviral engineering. After the first polyclonal stimulation, which facilitates CAR transduction, the use of aAPCs that express CAR target in the absence of an α-CD3 signal restimulates cells with functional levels of Bw6-specific CAR Tregs and enriches this population significantly, precluding the need for post-expansion purification and providing enough CAR Tregs to enable preclinical testing of organ transplants in NHPs.

A major unexpected finding of our study is the narrow persistence of CAR Tregs in peripheral circulation and their long-term persistence in the bone marrow. Others have labeled polyclonal NHP Tregs with the proliferation dye carboxyfluorescein succinimidyl ester (CFSE) as a more sensitive way to identify infused cells,^30^^,^^33^^,^^34^^,^^58^ enabling *in vivo* detection up to 100 days in peripheral blood. However, “CFSE toxicity” renders cells viable but less able to proliferate.^63^ Our method of identifying CAR Tregs via tetramer binding is compromised by rapid receptor internalization upon activation^64^^,^^65^ and a decrease in CAR MFI in the absence of signaling (Figure 6), reducing sensitivity and increasing the limit of detection to ∼0.5% of CD4^+^ FoxP3^+^ cells versus ∼0.01% with CFSE.^33^ Thus, given the distinct sensitivities of methods to detect expanded Tregs in the peripheral blood, it is unclear whether the cells we infused persisted for less time in the periphery in the absence of antigen than in previous studies. In humans, polyclonal Tregs labeled with deuterium exhibited a similar, biphasic decay as seen here yet remained in circulation 180 days after transfer to kidney transplant recipients^12^ and >1 year following transfer in new onset type 1 diabetics.^66^

Adoptively transferred Bw6-specific CAR Tregs preferentially trafficked to and persisted in recipient bone marrow for >1 month. In humans and monkeys, T cells represent a minor fraction (3%–8%) of the total nucleated cells within the bone marrow with the relative abundance of Tregs and memory cells being enriched relative to their percentages in the peripheral blood.^67^^,^^68^ Bone marrow T cells express high levels of CXCR4, whose ligand CXCL12 is highly expressed within the bone marrow, which is thought to be a key player mediating the infiltration of Tregs.^69^ Elegant bar coding and parabiosis studies established that bone marrow is a temporary holding spot for T cells as they migrate throughout the body.^70^, ^71^, ^72^ Thus, it would be reasonable to assume that, once MHC mismatched organs are transplanted into the recipient, recognition of the target antigen by Tregs emigrating from the bone marrow would increase CAR Treg presence adjacent to transplanted tissue. Since bone marrow is only connected to the blood, without lymphatic access,^67^ this may facilitate the ability of the CAR Tregs to find the intended target tissue. Indeed, tumor-antigen-specific Tregs were shown to mobilize from the bone marrow toward tumor-associated CCL2 and provide a dominant dampening of the effector response,^73^ and in our study, the CAR Tregs migrated to the site of Bw6^+^ islet. Recent studies have demonstrated the feasibility of the bone marrow space as an alternative site for pancreatic islet grafts.^60^^,^^74^^,^^75^ However, in a pilot trial in type 1 diabetes (T1D) patients, diabetic autoimmune attack drove the loss of islet function within 4 months post-transplant, demonstrating the lack of immune privilege and illuminating the need for further local immunosuppression.^75^ The addition of CXCR4^+^ alloantigen-specific adoptive Tregs, which we show here naturally traffic to the bone marrow and become activated by allograft, could deliver targeted immune suppression to protect bone marrow transplanted islets. Targeted irradiation of transplantation site creating space within the marrow,^76^ a strategy that also improves engraftment of CAR Teffs for cancer, could also synergize with alloantigen-specific CAR Treg therapy in clinical practice.

A phase I/II trial was initiated as the first-in-human clinical trial of alloantigen-specific CAR Tregs in 2019 (NCT04817774). The STEADFAST study will gauge the safety of HLA-A2-specific CAR Treg therapy following mismatched kidney allotransplantation. Human studies have also preceded NHP studies for using CAR T cells as a means to cure HIV infection,^77^ due to similar roadblocks that have recently been overcome.^78^ Now that these manufacturing issues have been overcome in both disease settings, we suggest that the side-by-side human and NHP clinical trials be performed to better understand the fidelity of NHP models for non-cancer CAR T cell therapies and perhaps to provide better mechanistic data by obtaining NHP samples and tissues that would be impossible to obtain in human studies. Overall, our aAPC-based expansion protocol combined with modified HIV lentiviral vectors have overcome the roadblocks associated with manufacture of engineered NHP Tregs, enabling the optimization of timing, dosage, and induction and maintenance immunosuppression with a human/NHP cross-reactive alloantigen-specific CAR construct to improve the chances of clinical success.

### Limitations of this study

The limitations of this study are that we only evaluated one allospecific CAR (Bw6 specific) and that the transplant experiment performed in Figure 7 is an n = 1 experiment. The full ramifications of concurrent CAR Treg and islet transplantation must be ascertained in future, larger cohorts of animals, which will include control animals not receiving Tregs.

## STAR★Methods

### Key resources table

**Table:** 

REAGENT or RESOURCE	SOURCE	IDENTIFIER
**Antibodies**		

CD4-BV421	Biolegend	Cat#317434; Clone: OKT4; RRID:AB_2562134
CD4-AF488	Biolegend	Cat#317420; Clone: OKT4; RRID:AB_571939
CD4-PerCP-Cy5.5	Biolegend	Cat#317428; Clone: OKT4; RRID:AB_1186122
CD4-BV605	Biolegend	Cat#317438; Clone: OKT4; RRID:AB_11218995
CD8-BV510	Biolegend	Cat#301048; Clone: RPA-T8; RRID:AB_2561942
CD25-BV421	Biolegend	Cat#302630; Clone: BC96; RRID:AB_11126749
CD127-PE	Biolegend	Cat#351301; Clone: A019D5; RRID:AB_10720815
CXCR4-APC	Biolegend	Cat#306510; Clone: 12G5; RRID:AB_314616
CD45RA-FITC	BD Biosciences	Cat#556626; Clone: 5H9; RRID:AB_396498
CD3-PerCP-Cy5.5	BD Biosciences	Cat#552852; Clone: SP34-2; RRID:AB_394493
Goat α-Mouse IgG F(ab)_2_-AF488	Jackson Immunoresearch	Cat#114-454-072
Fixable Viability Dye eFluor 780	eBioscience	Cat#65-0865-18
CTLA-4-BV786	BD Biosciences	Cat#563931; Clone: BNI3; RRID:AB_2738491
Helios-PE/Cy7	Biolegend	Cat#137236; Clone: 22F6; RRID:AB_2565990
FoxP3-PE/Dazzle 594	Biolegend	Cat#320126; Clone: 206D; RRID:AB_2564025
HLA-B∗07:02 HIV nef TPGPGVRYPL-PE	MBL International	Cat#TS-M054-1
HLA-A∗02:01 HIV gag SLYNTVATL-APC	MBL International	Cat#TB-M027-2
IL2-APC	BD Biosciences	Cat#554567; Clone: MQ1-17H2; RRID:AB_398571
TNFα-PE/Cy7	BD Biosciences	Cat#557647; Clone: MAB11; RRID:AB_396764
IFNγ-FITC	BD Biosciences	Cat#552882; Clone: 4S.B3; RRID:AB_394511
MIP1β-PerCP-Cy5.5	BD Biosciences	Cat#560688; Clone: D21-1351; RRID:AB_1727567
IL-10-PE/Dazzle594	Biolegend	Cat#501426; Clone: JES3-9D7; RRID:AB_2566744
LAP-APC	Biolegend	Cat#349706; Clone: TW5-6H10; RRID:AB_10680787
CD25-PE/Dazzle 594	Biolegend	Cat#302646; Clone: BC96; RRID:AB_2734260
CD8-PE	Biolegend	Cat#301008; Clone: RPA-T8; RRID:AB_314126
CD86-BV421	Biolegend	Cat#374212; Clone: BU63; RRID:AB_2728394
Streptavidin-PE	BD Biosciences	Cat#554061; RRID:AB_10053328
Cynomolgus CD3 epsilon protein	ACRO Biosystems	Cat#CDE-C5254
Biotinylated Human CD3 epsilon protein	ACRO Biosystems	Cat#CDE-H82E1
His Tag-AF647	Biolegend	Cat#652513; Clone: J099B12; RRID:AB_2716153
Active Caspase-3-BV650	BD Biosciences	Cat#564096; Clone: C92-605; RRID:AB_2738589

**Biological samples**		

Fetal Bovine Serum	Avantor Seradigm	Cat#97068-085
Human Serum Albumin	CSL Behring	N/A

**Chemicals, peptides, and recombinant proteins**		

Penicillin-Streptomycin	Gibco	Cat#15140122
Proleukin (aldesleukin)	Clinigen	N/A
Percoll	Millipore Sigma	Cat#P4937
ACK Lysing Buffer	Quality Biological	Cat#118-156-101
Phorbol 12-myristate 13-acetate (PMA)	Sigma Aldrich	Cat#P1839
Ionomycin	Sigma Aldrich	Cat#407950
GlutaMAX Supplement	Gibco	Cat#35050061
HEPES	Gibco	Cat#15630080
RPMI 1640	Gibco	Cat#11875085
Normocin	Invivogen	Cat#ant-nr-1
OpTmizer T Cell Expansion Medium	Gibco	Cat#A1048501
Rapamycin (Rapamune)	Pfizer	N/A
Rapamycin	LC Laboratories	R-5000
Streptozotocin (Zanosar)	Pharmacia & Upjohn	N/A

**Critical commercial assays**		

FlowPRA single antigen HLA Class I Beads	One Lambda	Cat#FL1HD02, Group 2
FlowPRA single antigen HLA Class I Beads	One Lambda	Cat#FL1HD08, Group 8
True-Nuclear Transcription Factor Buffer Set	Biolegend	Cat#424401
T Cell Activation/Expansion Kit, non-human primate	Miltenyi Biotec	Cat#130-092-919
CellTrace Violet	Molecular Probes	Cat#C34557
CellTrace CFSE	Molecular Probes	Cat#C34554
CellTrace Far Red	Molecular Probes	Cat#C34564
CountBright Absolute Counting Beads	Invitrogen	Cat#C36950
GolgiPlug Protein Transport Inhibitor	BD Biosciences	Cat#555029
DNeasy Blood & Tissue Kit	Qiagen	Cat#69504
Monkey FOXP3 Assay	Epigendx	Cat#ADS783-FS4
Fixation Medium A	Invitrogen	Cat#GAS001S100
Fixation Medium B	Invitrogen	Cat#GAS001S100
Lipofectamine 2000	Invitrogen	Cat#11668027
C-peptide specific ELISA	ARUP Laboratories	N/A
Insulin (Humulin R)	Eli Lilly and Company	N/A
Insulin (Glargine)	Eli Lilly and Company	N/A
CMRL 1066	Corning	Cat#98-304-CV
Valgancyclovir (Valcyte)	Genentech	N/A

**Experimental models: Cell lines**		

K-562	ATCC	CCL-243

### Resource availability

#### Lead contact

Further information and requests for resources and reagents should be directed to and will be fulfilled by the lead contact, James L. Riley (rileyj@upenn.edu).

#### Materials availability

This study generated three lentiviral transfer plasmids encoding CARs recognizing Bw6, HLA-A2, and pan-primate CD3, and one aAPC expressing pan-primate CD3 and human CD86. All are available from the Lead contact with a completed Materials Transfer Agreement.

### Experimental model and subject details

#### Animals

Adult and juvenile Mauritian origin *Cynomolgus macaques* were used for these studies from Charles River Primates; Bioculture Group; Alpha Genesis Inc. All animals were negative for B virus, simian T-lymphotropic virus, simian retrovirus, SIV, simian varicella virus, and malaria. All macaques were housed at the University of Pennsylvania’s Laboratory Animal Resources (ULAR) (Philadelphia, PA). The University of Pennsylvania holds a current USDA assurance and is an AAALAC-accredited institution. All procedures were performed in accordance with NIH guidelines for the care and use of primates and approved by the University of Pennsylvania Institutional Animal Care and Use Committee.

#### aAPC and cell line culture

All K562-based aAPCs were grown in RPMI medium (Gibco) supplemented with 10% Fetal Bovine Serum (FBS), 1x GlutaMAX (Gibco), 10 mM HEPES (Gibco), and 1x Penicillin-Streptomycin (Gibco) at 37°C with 5% CO_2_. Cells were maintained at 25k-50k cells/mL every 2–3 days. 293T cells were used for lentiviral manufacturing by culture in the same complete RPMI medium at 37°C. To generate our NHP aAPCs, we dual transduced parental K562 cells with lentiviruses encoding pan-primate α-CD3ζ CAR and full length human CD86. Cells were single cell sorted on a FACS Jazz cell sorter (BD) into a 96 well round bottom plate containing complete RPMI and 100 μg/mL Normocin (Invivogen). After 3 weeks of incubation at 37°C with 5% CO_2_, individual clonal populations were screened for transgene expression and T cell stimulatory function. The most stimulatory clone was then further expanded, γ-irradiated (100 gy), and frozen for future use.

### Method details

#### CAR plasmid and lentiviral vector generation

α-Bw6 antibody sequence (Clone: FD125) was provided by One Lambda (Thermo Fisher, West Hills, CA), adapted into an scFv, and cloned upstream of human CD8 hinge, CD28 transmembrane, and CD28 and CD3ζ intracellular signaling domains in a pTRPE transfer plasmid.^79^^,^^80^ HLA-A2 CAR was similarly constructed from an HLA-A2/A68/A69 specific antibody.^49^ Plasmid DNA was extracted from Stbl3 *E. coli*. 27 μg pTRPE transfer plasmid were mixed with 3 μg codon-optimized Cocal-g plasmid (ATUM, Newark, California)^81^ or 7 μg of VSV-g, 18 μg HIV-SIV chimeric gag-pol packaging plasmid that was constructed by replacing the entire HIV p24 portion of HIV gag with p24 derived from SIVmac239,^52^^,^^53^ 18 μg HIV_REV_ expression plasmid (pTRP Rev), and transfected using Lipofectamine 2000 (Invitrogen) into 293T cells. 24- and 48-h supernatants were ultracentrifuged for 2.5 h at 25000 rpm at 4°C, split into four aliquots, and stored at −80°C until use.

#### Human Teff isolation, transduction, and expansion

De-identified, purified human T cells were obtained from the Human Immunology Core at the University of Pennsylvania. T cells were stimulated with γ-irradiated (100 gy.) K562 cells expressing OKT3 scFv and human CD86 at a 1:2 ratio in OpTmizer media (Gibco) supplemented with 1x GlutaMAX (Gibco), 10 mM HEPES (Gibco), 1x Penicillin-Streptomycin (Gibco), and 100 IU/mL interleukin-2 (Proleukin, Clinigen). 100–200 μL of concentrated lentiviral vector supernatants per 500k T cells was added 24 h after initial stimulation. Cells were replenished every other day with culture media and IL-2 as needed.

#### *C.ynomolgus macaque* PBMC isolation and Treg sorting

Venous blood was diluted 1:1 with PBS and subjected to density gradient centrifugation over a 60% Percoll solution (Millipore Sigma) for 30 min at 2000 RPM. PBMCs at the interface were transferred to a new tube to undergo ACK lysing (Quality Biological) before staining with CD4-BV421 (Clone: OKT4), CD8α-BV510 (RPA-T8), CD25-PE/Dazzle594 (BC96), CD127-PE (A019D5), and CD45RA-FITC (5H9) and sorting of CD4^+^ CD8^−^ CD25^+^ CD127^-/lo^ CD45RA^+^ cells on a FACSJazz flow cytometer (BD).

#### NHP T cell transduction and expansion

Sorted NHP T cells were co-cultured with γ-irradiated (100 gy.) K562s bearing a pan-primate α-CD3 scFv and CD86 at a 1:1 ratio in RPMI supplemented with 10% FBS, 10 mM HEPES (Gibco), 1x GlutaMAX (Gibco), 1x Penicillin-Streptomycin (Gibco), and 300 IU/mL (Tregs) or 100 IU/mL (Teffs) IL-2 (Proleukin, Clinigen). On day 2 following stimulation, lentiviral vector supernatant was added to the cells. At days 7 and 14, Tregs were re-stimulated by co-culture with irradiated K562s expressing HLA-B7 (Bw6^+^) and human CD86. Cells were fed with media and IL-2 on days 3, 5, 9, 11, 13, 16, and 18. Where indicated, T Cell Activation/Expansion Kit, non-human primate α-CD3/28 or α-CD2/3/28 beads (Miltenyi Biotec) were used for T cell stimulation at a ratio of 2 beads for every T cell and grown as per manufacturer’s instructions.

#### Antigen non-specific suppression assay

Allogeneic *Cynomolgus macaque* PBMCs were labeled with 5 μM CellTrace Violet or CellTrace CFSE (Molecular Probes) as per manufacturer’s instructions and co-cultured with either Tregs or Teffs and α-CD3/28 beads (Miltenyi Biotec) in RPMI growth medium without cytokines at a ratio of 1 bead per 2 PBMCs. After 4–5 days, cells were stained with CD4-BV421 or CD4-AF488 (OKT4), CD8-BV510 or CD8-PE (RPA-T8), and Fixable Viability Dye eFluor 780 and analyzed by flow cytometry. Percent suppression was calculated as [1- (number of CD8^+^ T cell divisions per cell in suppressed condition/number of CD8^+^ T cell divisions per cell in unsuppressed condition)] x 100.

#### Mixed lymphocyte reaction

Sorted Bw6^-^ NHP T effs labeled with 5 μM CellTrace Violet were co-cultured with allogeneic, γ-irradiated PBMCs (30 gy.) at a 1:2 ratio plus CAR Tregs labeled with 5 μM CellTrace Far Red for 7 days. Cells were then stained with CD4-AF488 (OKT4) and CD8-BV510 (RPA-T8) antibodies and Fixable Viability Dye eFluor 780 and analyzed by flow cytometry. Percent suppression was calculated as above.

#### LAP assay

Tregs were co-cultured with indicated target cells at a ratio of 1 Treg per 2 target cells in complete RPMI with 300 IU/mL IL-2. Following 24 h of co-culture, Tregs were stained with CD4-BV421 (OKT4), LAP-APC (TW4-6H10, Biolegend), and Fixable Viability Dye eFluor 780 (eBioscience) and analyzed by flow cytometry.

#### Killing assay

CAR Tregs or Teffs were co-cultured at the indicated ratios with on-target K562.A2.B7 cells and off-target K562.A2 cells, one of which was labeled with 5 μM CellTrace Violet. Both cell lines were previously transduced to express mCherry to differentiate them from T cells. After 24 h of co-culture, CountBright Absolute Counting Beads (Invitrogen) were added, and cells were analyzed by flow cytometry without washing.

#### FlowPRA assay

10,000 transduced T cells were incubated with FlowPRA single antigen HLA Class I beads (Group 2 or 8, One Lambda) for 30 min at RT with gentle shaking, washed, and fixed with 2% paraformaldehyde. Unbound FlowPRA beads were gated based on SSC/FSC, and resulting histograms were inspected for a decrease in bead number versus control cells to indicate binding to T cells.^41^

#### Flow cytometry

Washed cells were stained in PBS + 2% FBS + 1 mM EDTA with antibodies from Biolegend: CD4-BV421/AF488/PerCP-Cy5.5/BV605 (Clone: OKT4), CD8α-BV510 (RPA-T8), CD25-BV421 (BC96), CD127-PE (A019D5), CXCR4-APC (12G5); BD: CD45RA-FITC (5H9), CD3-PerCP-Cy5.5 (SP34-2); or Jackson Immunoresearch: Goat α-Mouse IgG F(ab’)_2_-AF488 (Jackson Immunoresearch) with Fixable Viability Dye eFluor 780 (eBioscience). All extracellular stained cells were fixed in 2% paraformaldehyde before analysis. The TrueNuclear Transcription Factor Buffer Set (Biolegend) was used as per manufacturer’s instructions for staining with FoxP3-PE/Dazzle594 (206D, Biolegend), Helios-PE/Cy7 (22F6, Biolegend) or tetramer (HLA-B∗07:02 HIV nef TPGPGVRYPL-PE or HLA-A∗02:01 HIV gag SLYNTVATL-APC (MBL International). Whole blood and bone marrow samples were ACK lysed and blocked with Fcr Blocking Reagent (Miltenyi Biotec) before staining. Spleen, liver, and lymph nodes were mechanically disrupted through a 70 μm strainer to isolate single cell suspension. All cells were analyzed on a BD Fortessa flow cytometer.

#### Intracellular cytokine staining

Transduced T cells were co-cultured with target cells at a 1:2 ratio or with 3 μg/mL PMA (Sigma Aldrich) and 1 μg/mL Ionomycin (Sigma Aldrich) for 5 h in the presence of GolgiPlug Protein Transport Inhibitor (BD Biosciences). Cells were then stained for surface markers, fixed with Fixation Medium A (Thermo Fisher Scientific), washed, and stained with IL2-APC (MQ1-17H2, BD), TNFα-PE-Cy7 (MAB11, BD), IFNγ-FITC (4S.B3, BD), MIP1β-PerCP-Cy5.5 (D21-1351, BD), and IL-10-PE/Dazzle 594 (JES3-9D7, Biolegend) antibodies in Fixation Medium B (Thermo Fisher Scientific) for 20 min. Following a final wash, cells were analyzed on a BD Fortessa flow cytometer.

#### Bisulfite pyrosequencing

Genomic DNA was isolated with DNeasy Blood & Tissue Kit (Qiagen). Pyrosequencing of bisulfate converted DNA was performed by Epigendx (Monkey FOXP3 assay, ADS783-FS4) on chrx:47113171-47113104.

#### Infusion of Tregs

Recipient was sedated with dexmedetomidine and ketamine and maintained under anesthesia using isoflurane with 100% oxygen. Tregs were resuspended in 50 mL of Hank’s Balanced Sodium Solution + 0.1% human serum albumin and infused into the saphenous or cephalic vessels with a 21 gauge intracatheter. Infusions were delivered over 15–20 min. Animals were given oral rapamycin (Rapamune, 2mg tablets, Pfizer) or injected subcutaneously with a sterile injectable rapamycin solution of 1 mg/mL (LC Laboratories) aiming for a target blood level of 10–20 ng/mL. Rapamycin dose was modified as guided by blood levels. Recombinant IL-2 (Proleukin, Clinigen) was injected s.c at 1 million units/m^2^.

#### Induction of hyperglycemia

Transplant recipient was fasted overnight with access to water. The following day, the recipient was sedated with ketamine and dexmedetomidine cocktail and provided oxygen. Streptozotocin (STZ, Zanosar, Pharmacia & Upjohn, Peapack, NJ) was reconstituted in 5.0 ml of 0.9% NaCl and an 85 mg/kg IV slow push dose was administered through the saphenous vein over 5 min followed by infusion of 10 mL of physiologic saline. The recipient was pre-hydrated with 0.9% NaCl (60 mL/kg) IV prior to STZ injection. Blood glucose was monitored closely after being rendered diabetic twice daily via tail or heel sticks. When three consecutive glucose assessments 8 h apart exceeded 250 mg/dL and c-peptide levels were <0.5 ng/ml (C-peptide specific ELISA assay, ARUP Laboratories, Salt Lake City, UT) the recipient was considered to be in a stable diabetic state. Insulin (Humulin R and/or Glargine, Eli Lilly and Company) was administered on a sliding scale regimen to achieve a blood glucose level <200 mg/dL. Recurrence of the diabetic state was defined as two consecutive non-fasting blood glucose measurements exceeding 150 mg/dL at which point insulin was resumed for the wellbeing of the animal.

#### Islet transplant

With the recipient monkey under anesthesia, the monkey was placed on ventral recumbency and the hips slightly elevated. The iliac crest area was shaved and aseptically prepped. The iliac cortex and subcutaneous tissues were blocked locally with bupivacaine and a 16 gauge Jamshidi needle was used to enter the marrow cavity for islet infusion. Islets were infused with heparinized CMRL 1066 media (35 units/kg) (Corning) containing isolated islets (2–3 × 10^4^ islet equivalents/kg recipient body weight) by gravity over a period of approximately 10 min into marrow cavity.

#### Donor pancreatectomy and islet isolation

This procedure was performed as previously published.^82^ Briefly, the donor pancreatectomy was performed one day prior to the intramarrow islet transplant. The donor was sedated with dexmedetomidine and ketamine. Islets were purified from the digested pancreas using a three-layer, discontinuous Euroficoll gradient and a COBE blood cell processor (Cobe Laboratories, Lakewood, CO). Samples were collected from different layers after purification for purity. Final samples were stained with dithizone, counted manually, and sized using a formula to calculate islet number and islet equivalents (IEQ) based on their diameter. Islet purity was >85%. The isolated islets were cultured overnight in CMRL 1066 (Corning) containing 10% heat inactivated FBS at 25–28°C in 95% O_2_, 5% CO_2_ and infused on the recipient as described.

#### Transplant preparatory/immunosuppressive regime

Transplant recipient received CMV prophylaxis with Valganciclovir (Valcyte, Genentech) at a dose of 12.5 mg/kg once daily for two weeks starting at the day of transplantation and discontinued if CD8 T cell counts were >500 cells/μL. Rapamycin was given orally (2 mg tablets Rapamune, Pfizer) or subcutaneously from a sterile injectable solution (LC Laboratories) at a concentration of 1 mg/mL starting 6 days before transplantation and lasting 20 days. Rapamycin dose was tailored to achieve blood levels of 10–20 ng/mL. Human recombinant IL-2 (Proleukin, Clinigen) at 1 million units/m^2^ was injected subcutaneously starting 6 days before transplant and lasting 4 days. After any procedure, all animals were given buprenorphine SR which provided 72 hours of analgesia. Animals were observed daily, and if pain was suspected, they were given additional analgesics (meloxicam and/or additional opioids) for comfort.

### Quantification and statistical analysis

#### Flow cytometry data analysis

Flow cytometry was analyzed using FlowJo software with fluorescence minus one (FMO) gating or fully stained negative samples used to define positive populations.

#### Statistical analysis

Bar and line graphs were generated using GraphPad Prism depicting mean ± SEM error bars. Statistical details of experiments and values of n can be found in figure legends.

## Data Availability

All data reported in this paper will be shared by the lead contact upon request. This paper does not report the original code. Any additional information required to reanalyze the data reported in this paper is available from the lead contact upon request.

## Supplemental information

Document S1. Figures S1–S6

Document S2. Article plus supplemental information
