# Supplementary material for: Trafficking and persistence of alloantigen-specific chimeric antigen receptor regulatory T cells in Cynomolgus macaque
Source: Cell Rep Med. 2022 May 11;3(5):100614. doi: 10.1016/j.xcrm.2022.100614 (PMC9133392; doi:10.1016/j.xcrm.2022.100614)
Supplement: Document S1. Figures S1–S6 [file mmc1.pdf]

Cell Reports Medicine, Volume *3*

Supplemental information

Trafficking and persistence of alloantigen-specific chimeric antigen receptor regulatory T cells in *Cynomolgus macaque*

Gavin I. Ellis, Kimberly E. Coker, Delaine W. Winn, Mosha Z. Deng, Divanshu Shukla, Vijay Bhoj, Michael C. Milone, Wei Wang, Chengyang Liu, Ali Naji, Raimon Duran-Struuck, and James L. Riley

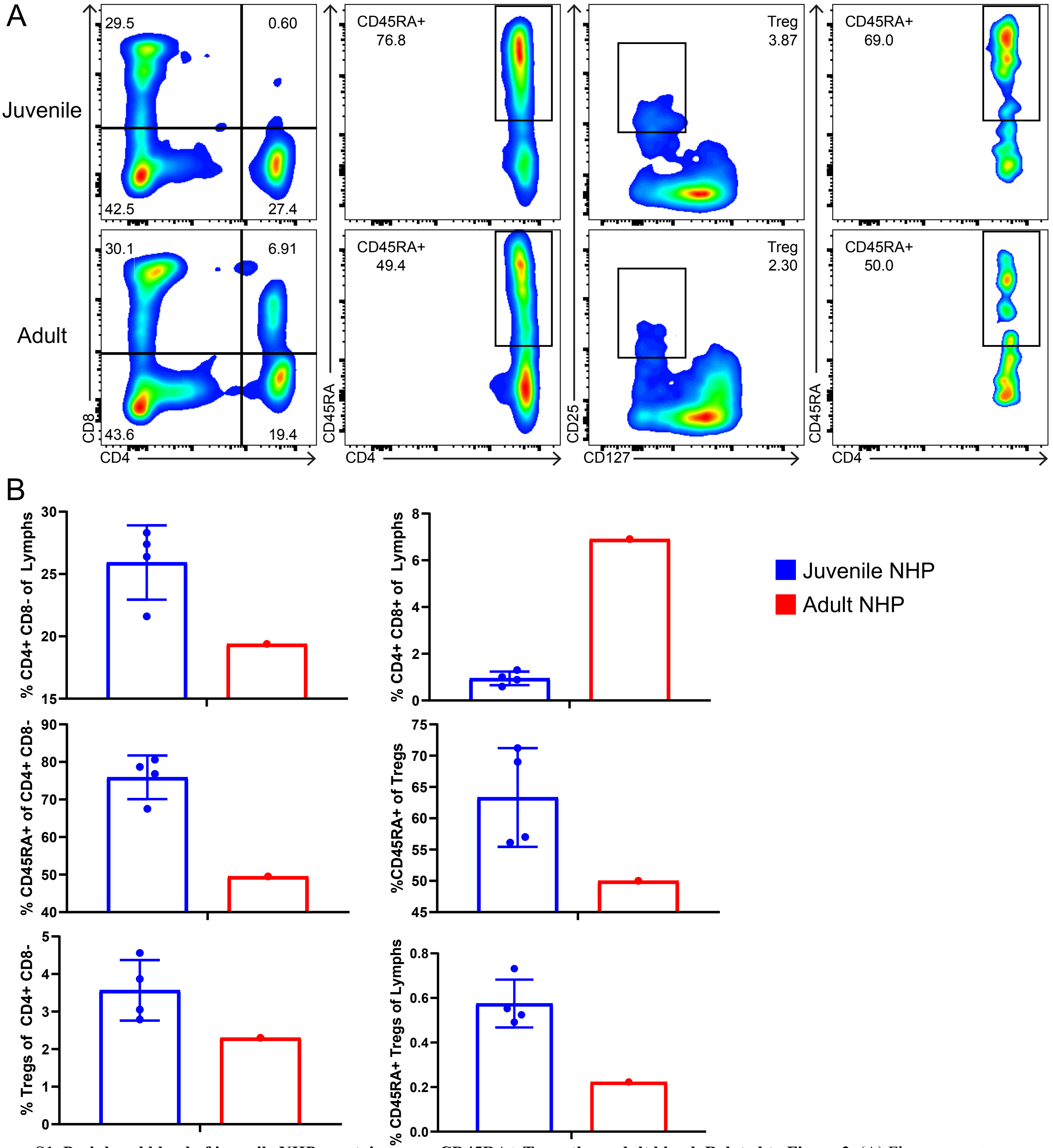

Figure S1. Peripheral blood of juvenile NHPs contains more CD45RA+ Tregs than adult blood, Related to Figure 2. (**A**) Flow cytometry phenotype of peripheral blood from juvenile (top) or adult (bottom) NHPs. (**B**) Summary of peripheral blood flow phenotypes across 5 animals stratified by age.

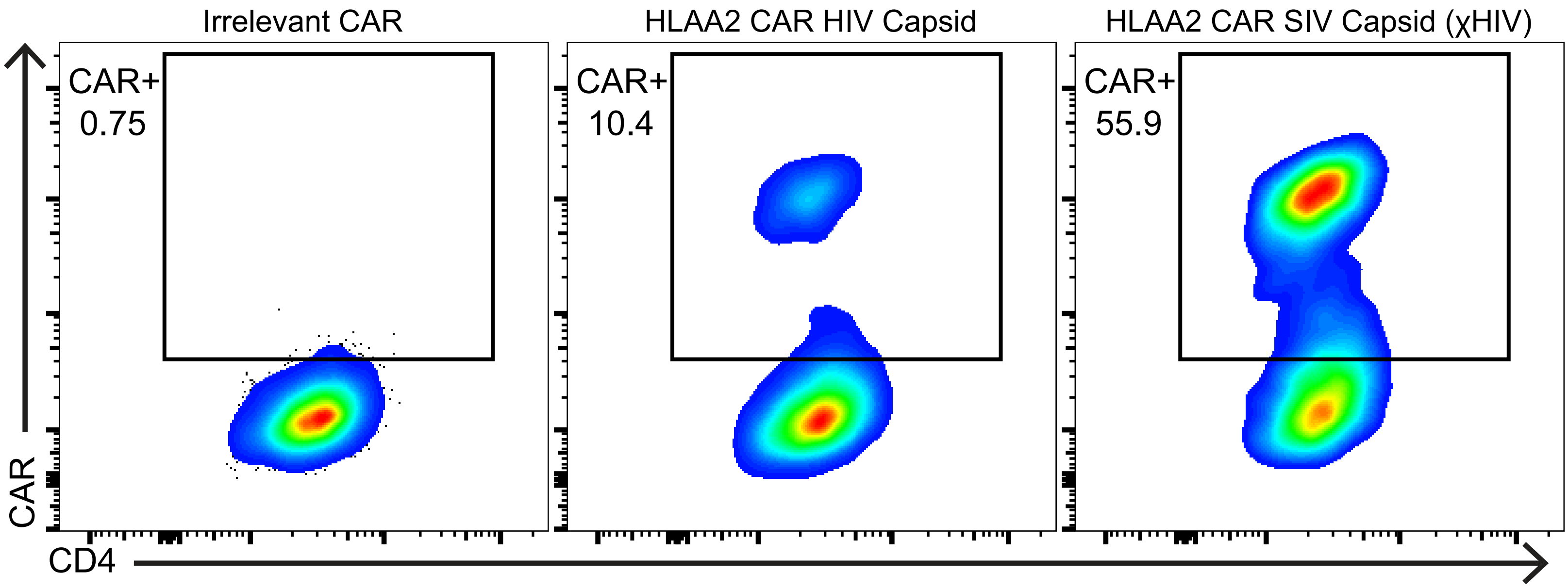

Figure S2. Lentiviral transduction with SIV capsid (χHIV) virus outperforms HIV capsid virus, related to Figure 2. *Cynomolgus macaque* T cells were transduced with HLAA2 CAR lentivirus containing HIV or SIV capsid and stained for CAR expression with HLA-A2 tetramer.

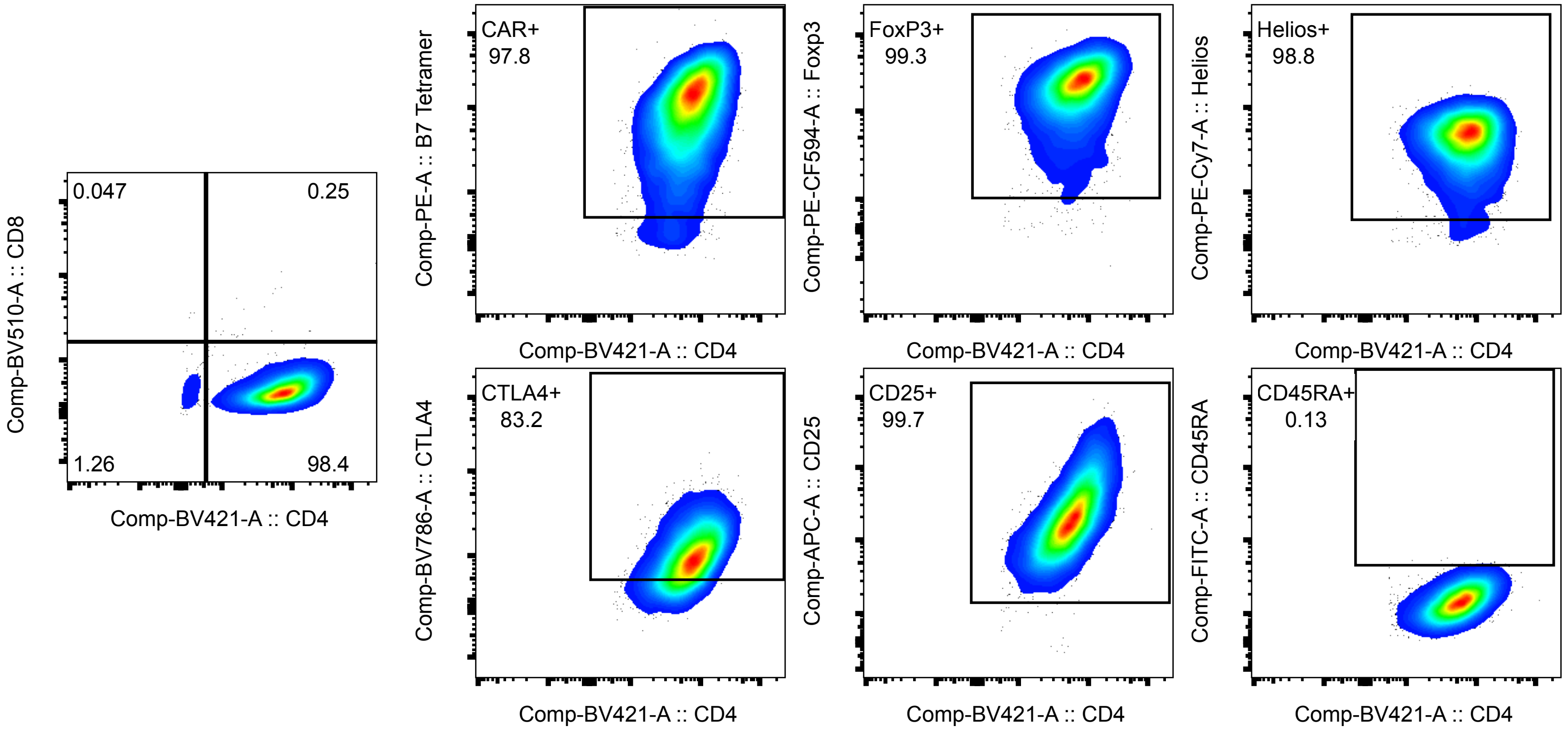

Figure S3. Expanded CAR Treg products express FoxP3, Helios, CTLA-4, and CD25, but lose CD45RA expression, Related to Figure 2. Flow cytometry phenotype of CAR Treg product following expansion. Data are representative of 8 similarly stained CAR Treg products.

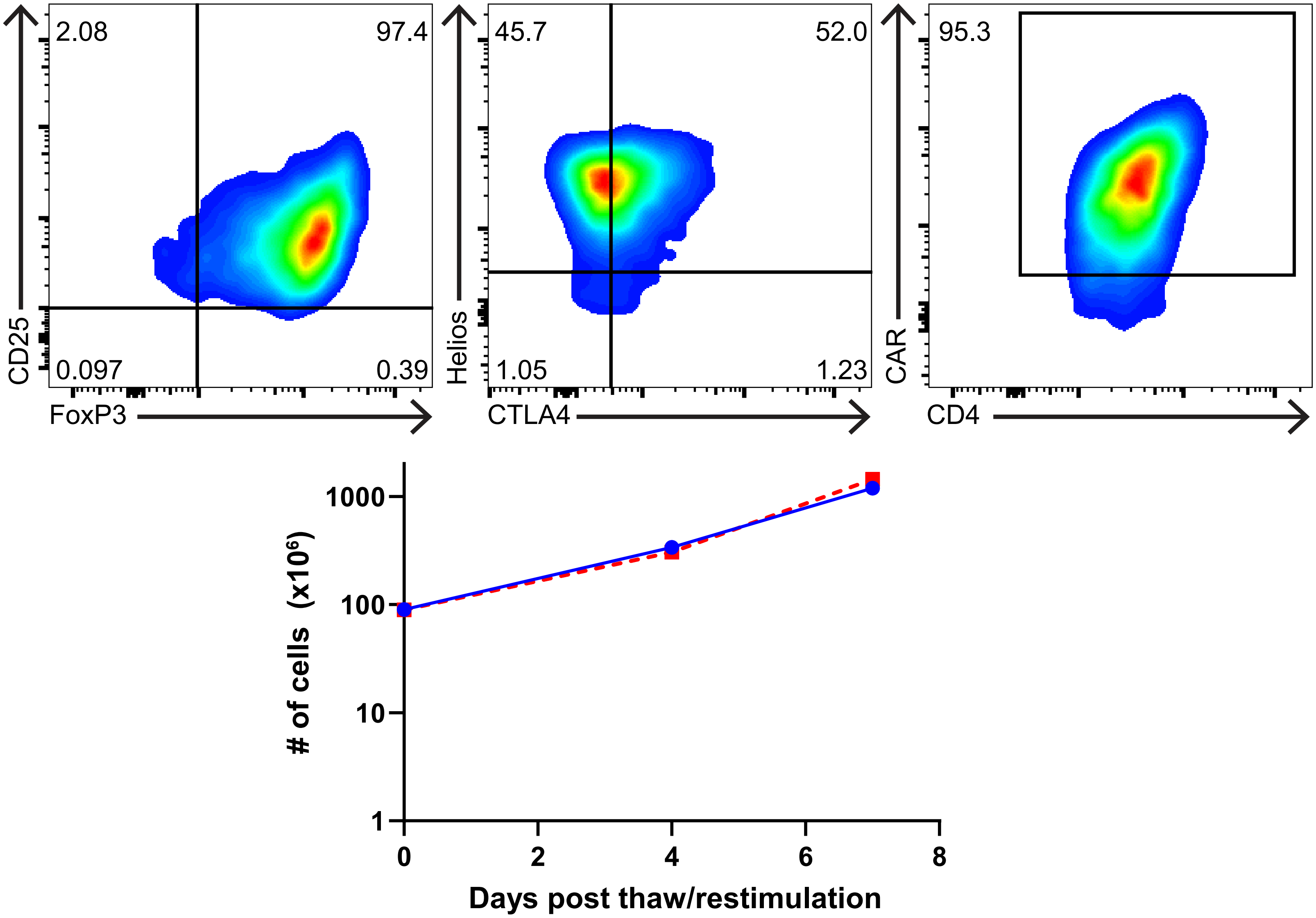

Figure S4. CAR Treg products survive cryopreservation and can be further expanded, Related to Figure 2. Flow cytometry phenotype (top) and expansion kinetics (bottom) 7 days following thawing and restimulation of cryopreserved CAR Treg product. Each line represents 1 of 2 separate expanded products. Gates are based on FMO controls.

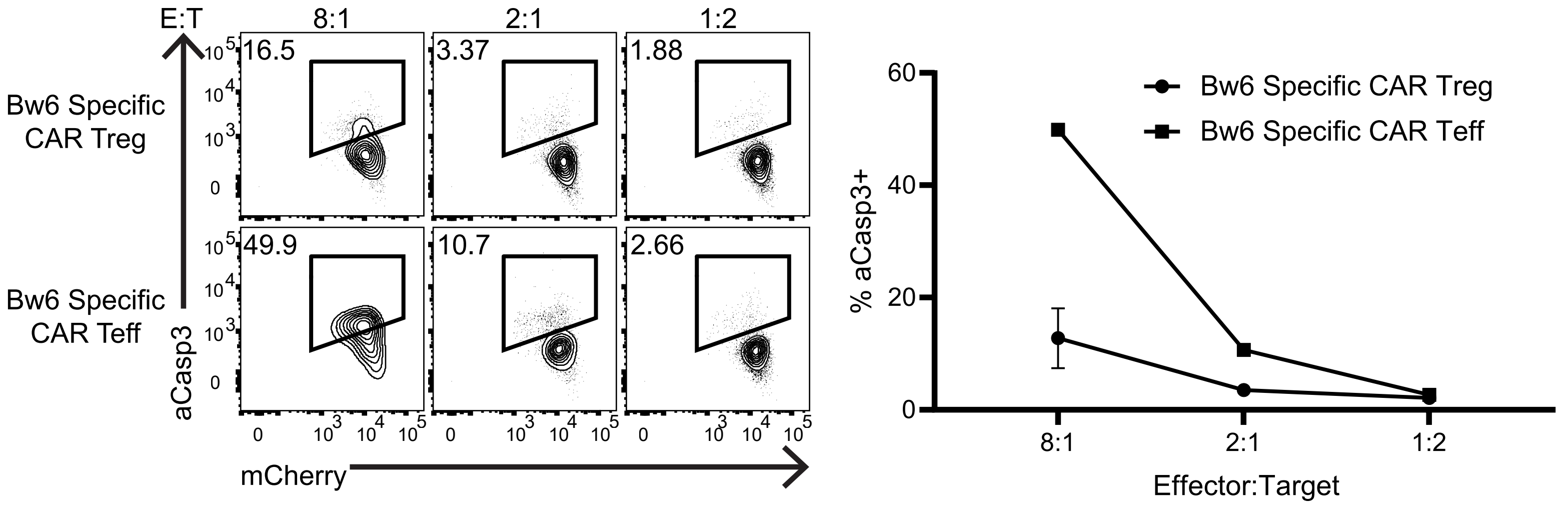

Figure S5. Bw6 specific CAR Tregs induce minimal active caspase 3 on target cells, Related to Figure 4. Bw6 specific CAR Tregs or CAR Teffs were mixed at the indicated effector to target ratio (E:T) with Bw6+ target aAPCs overnight before intracelluar staining with active caspase-3 antibody. Target cells were transduced with mCherry to differentiate them from CAR T cells.

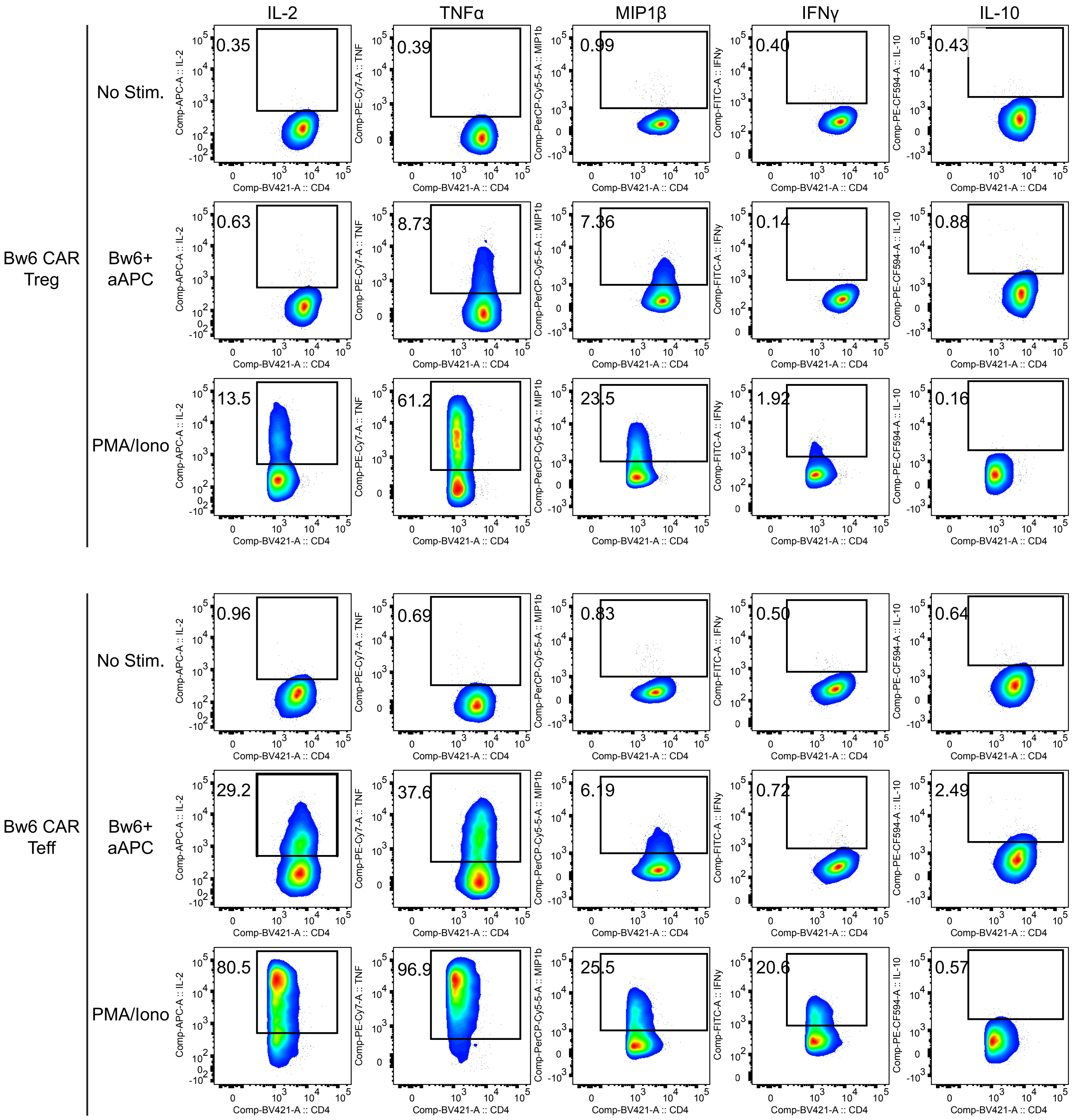

Figure S6. CAR Treg products secrete some TNFα and MIP1β, but no IL-2, IFNγ, or IL-10, Related to Figure 4. Flow plots displaying cytokine secretion following 5 hour co-culture between indicated cell types and Bw6+ aAPCs or PMA/ionomycin. Data are representative of 4 independent experiments.
